# Supplementary material for: The impact of occupational structures on ethnic and gendered employment gaps: An event history analysis using social security register data
Source: PLoS One. 2021 Apr 15;16(4):e0250398. doi: 10.1371/journal.pone.0250398 (PMC8049483; doi:10.1371/journal.pone.0250398)
Supplement: S1 Fig — (DOCX) [file pone.0250398.s001.docx]

S6 Figure: Ethnic occupational effects of the share of foreign employees among occupations on the employment incidence of native and immigrant workers


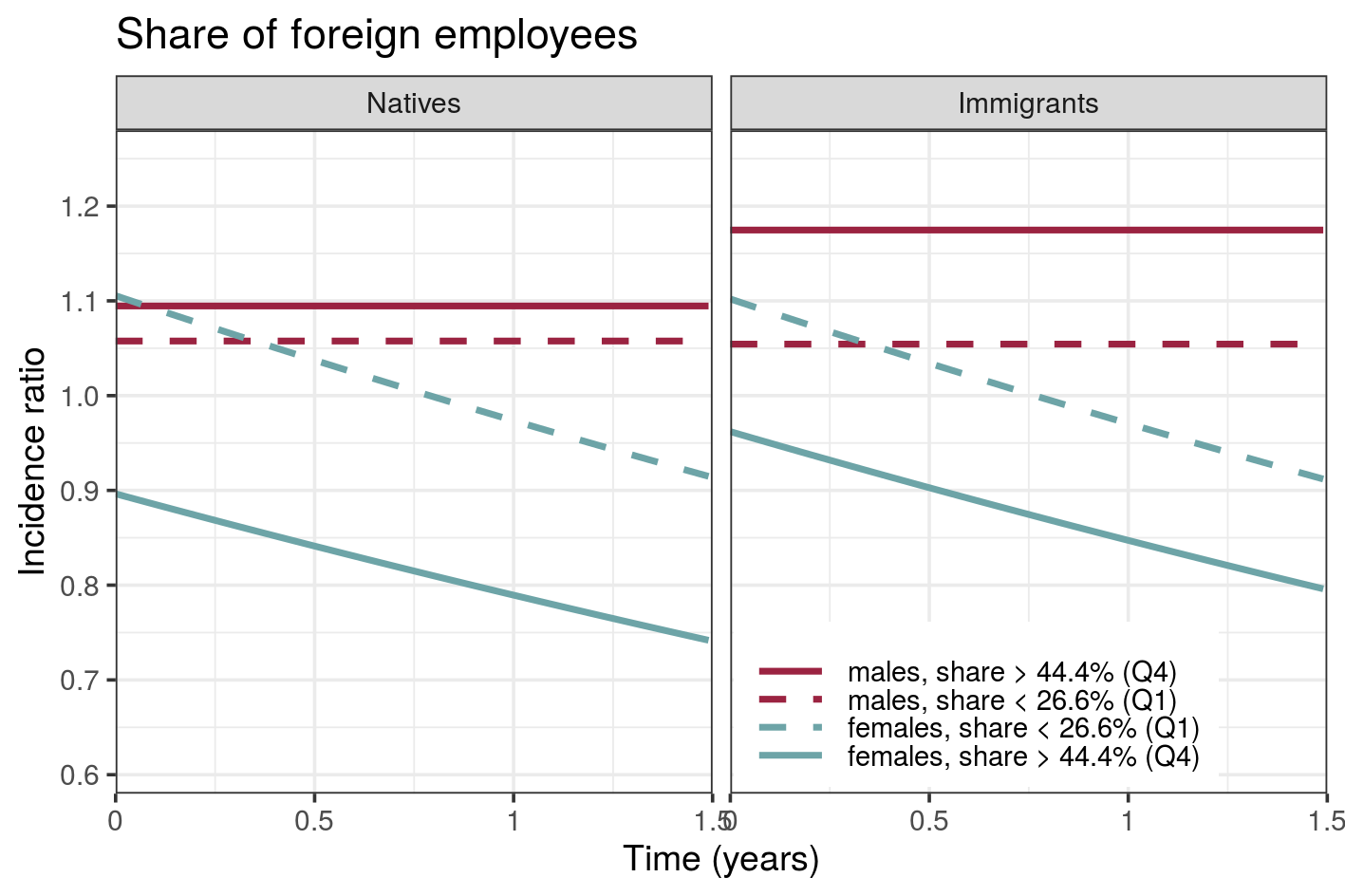


Source: LMDB. Predicted from an extended stratified cox proportional hazards model M3 (see Table 1), including an interaction between being foreign and the share of foreign employees in the aspired occupation, time to first employment spell of at least 91 days. Covariates were held constant at the following values: 21‑24 years of age, in the country for 14 years or longer, marital status single, lower secondary education credential, more than 180 days of work experience in 2013, and median values for other structural variables.
