# Supplementary material for: The impact of occupational structures on ethnic and gendered employment gaps: An event history analysis using social security register data
Source: PLoS One. 2021 Apr 15;16(4):e0250398. doi: 10.1371/journal.pone.0250398 (PMC8049483; doi:10.1371/journal.pone.0250398)
Supplement: S1 Table — (DOCX) [file pone.0250398.s002.docx]

S1 Table: Descriptive statistics of individual variables

|  | % female | % low  education^1^ | median days in employment (2013) | N |
| --- | --- | --- | --- | --- |
| *Austria* | *46.5%* | *33.8%* | *175* | *8,672* |
| *Former Yugoslav Republic (FYR)* | *46.0%* | *58.5%* | *153* | *3,834* |
| Serbia^2^ | 45.8% | 62.0% | 144 | 2,883 |
| Bosnia and Herzegovina | 45.4% | 44.0% | 179 | 443 |
| Croatia | 45.6% | 39.5% | 196 | 294 |
| North Macedonia | 50.0% | 66.8% | 159 | 214 |
| *Turkey* | *45.7%* | *62.6%* | *158* | *2,628* |
| *Eastern European Countries (EEU)* | *54.0%* | *49.9%* | *184* | *1,429* |
| Poland | 49.2% | 47.3% | 189 | 476 |
| Romania | 54.0% | 62.0% | 151 | 389 |
| Hungary | 54.4% | 35.8% | 238 | 193 |
| Slovakia | 64.8% | 48.7% | 198 | 193 |
| Bulgaria | 53.4% | 50.0% | 150 | 118 |
| Czech Republic | 58.3% | 41.7% | 201 | 60 |
| *Greater Middle East (GME)* | *28.9%* | *76.8%* | *81* | *900* |
| Afghanistan | 21.1% | 88.8% | 69 | 484 |
| Egypt | 39.3% | 61.5% | 93 | 122 |
| Iran | 27.7% | 58.4% | 109 | 101 |
| Iraq | 37.2% | 66.7% | 105 | 78 |
| Tunisia | 52.3% | 60.0% | 101 | 65 |
| Syria | 38.0% | 72.0% | 40 | 50 |
| **Total** | **46.0%** | **47.1%** | **163** | **17,463** |

Source: LMDB. ^1^No more than compulsory education (ISCED 0‑2 equivalent), ^2^including Montenegro and Kosovo.
