# Supplementary material for: The impact of occupational structures on ethnic and gendered employment gaps: An event history analysis using social security register data
Source: PLoS One. 2021 Apr 15;16(4):e0250398. doi: 10.1371/journal.pone.0250398 (PMC8049483; doi:10.1371/journal.pone.0250398)
Supplement: S2 Table — (DOCX) [file pone.0250398.s003.docx]

S2 Table: Descriptive statistics of structural occupational variables

| Occupational groups (ISCO-08 digits) | M-Index | Gini | share foreign | share female | N |
| --- | --- | --- | --- | --- | --- |
| Chief executives, senior officials and legislators (11) | 1.02 | 0.80 | 25.0% | 44.4% | 11 |
| Administrative and commercial managers (12) | 0.83 | 0.66 | 28.8% | 40.8% | 248 |
| Production and specialised services managers (13) | 0.47 | 0.55 | 34.8% | 30.1% | 51 |
| Hospitality, retail and other services managers (14) | 0.49 | 0.58 | 39.0% | 41.5% | 47 |
| Science professionals, mathematicians, architects (211-213, 216) | 1.29 | 0.75 | 25.8% | 46.0% | 205 |
| Engineering professionals (214, 214) | 1.45 | 0.83 | 32.5% | 18.0% | 51 |
| Medical doctors, Veterinarians (221, 225) | 3.63 | 0.99 | 20.2% | 65.5% | 15 |
| Nursing, midwifery and health professionals (222, 223, 226) | 2.03 | 0.86 | 28.8% | 83.0% | 59 |
| Teaching professionals (30) | 1.66 | 0.82 | 26.9% | 83.3% | 191 |
| Business and administration professionals (40) | 1.00 | 0.73 | 31.6% | 57.7% | 336 |
| ICT professionals (50) | 1.37 | 0.81 | 26.3% | 13.0% | 109 |
| Social and cultural professionals (262-265) | 1.61 | 0.79 | 31.1% | 65.7% | 106 |
| Legal professionals (261) | 3.95 | 0.97 | 32.7% | 60.0% | 94 |
| Physical and engineering science technicians (311) | 0.86 | 0.79 | 29.9% | 10.4% | 446 |
| Technicians and related associate professionals (312-315) | 0.53 | 0.62 | 33.3% | 12.9% | 43 |
| Health associate professionals (32) | 1.38 | 0.76 | 26.9% | 80.6% | 347 |
| Sales and purchasing agents and brokers (332) | 0.24 | 0.54 | 25.5% | 36.8% | 73 |
| Administrative and specialised secretaries (334) | 0.54 | 0.66 | 30.2% | 79.8% | 385 |
| Business, admin. associate prof. (331, 333, 335) | 0.52 | 0.67 | 16.0% | 64.6% | 209 |
| Legal, social, cultural and related associate prof. (34) | 0.48 | 0.50 | 22.9% | 57.6% | 318 |
| Information and communications technicians (35) | 0.74 | 0.75 | 24.1% | 6.8% | 288 |
| General office clerks (411) | 0.78 | 0.76 | 23.4% | 84.3% | 1,738 |
| Secretaries and keyboard operators (412, 413) | 0.57 | 0.69 | 28.0% | 78.9% | 110 |
| Customer services clerks (42) | 0.59 | 0.69 | 33.8% | 74.2% | 510 |
| Numerical and material recording clerks (43) | 0.37 | 0.63 | 20.1% | 60.4% | 167 |
| Other clerical support workers (44) | 0.37 | 0.58 | 41.6% | 53.8% | 20 |
| Personal service workers (511, 515, 516) | 0.22 | 0.50 | 56.3% | 43.8% | 118 |
| Cooks, waiters and bartenders (512, 513) | 1.16 | 0.78 | 61.1% | 57.9% | 1,322 |
| Hairdressers, beauticians and related workers (514) | 2.29 | 0.89 | 45.6% | 92.1% | 334 |
| Sales workers (52) | 0.71 | 0.72 | 43.6% | 74.4% | 3,246 |
| Personal care workers (53) | 0.67 | 0.59 | 50.0% | 89.1% | 529 |
| Protective services workers (54) | 0.40 | 0.60 | 35.6% | 28.0% | 181 |
| Skilled agricultural, forestry and fishery workers (6) | 1.09 | 0.68 | 25.6% | 28.5% | 109 |
| Building and related trades workers, excl. electricians (71) | 1.31 | 0.88 | 67.8% | 2.9% | 848 |
| Metal, machinery and related trades workers (72) | 1.21 | 0.85 | 34.4% | 3.2% | 533 |
| Handicraft and printing workers (73) | 0.82 | 0.67 | 61.0% | 31.0% | 25 |
| Electrical and electronic trades workers (74) | 1.56 | 0.90 | 45.2% | 4.7% | 472 |
| Food processing, wood, garment and related workers (75) | 1.15 | 0.72 | 37.7% | 39.1% | 233 |
| Stationary plant and machine operators (81) | 0.50 | 0.65 | 58.8% | 22.1% | 68 |
| Assemblers (82) | 0.34 | 0.61 | 27.3% | 36.2% | 20 |
| Drivers and mobile plant operators (83) | 0.58 | 0.71 | 52.3% | 4.5% | 393 |
| Cleaners and helpers (91) | 0.37 | 0.54 | 92.6% | 83.3% | 578 |
| Labourers, assistants, elementary workers (92-96) | 0.27 | 0.56 | 67.0% | 31.6% | 2,277 |

Source: LMDB and Austrian LFS. M-Index and Gini: Occupational closure measure.
