# Supplementary material for: The impact of occupational structures on ethnic and gendered employment gaps: An event history analysis using social security register data
Source: PLoS One. 2021 Apr 15;16(4):e0250398. doi: 10.1371/journal.pone.0250398 (PMC8049483; doi:10.1371/journal.pone.0250398)
Supplement: S3 Table — (DOCX) [file pone.0250398.s004.docx]

S3 Table: Stratified cox proportional hazards, time to recurrent employment events

|  | Origin countries  (initial model) | | | Origin countries net  (Model M4) | | |
| --- | --- | --- | --- | --- | --- | --- |
|  | b (SE) | hazard | | b (SE) | hazard | |
| *Gender (ref: male)* |  | |  |  |  | |
| Female | 0.07 (0.03) | | 1.07 | -0.05 (0.03) | | 0.95 |
| *Int*: female x time (/100 days) | -0.03 (0.01) | | 0.97 | -0.03 (0.01) | | 0.97 |
| *Countries (Ref: Austria)* |  | |  |  |  | |
| Serbia | -0.10 (0.03) | | 0.91 | -0.03 (0.02) | | 0.97 |
| Turkey | -0.15 (0.04) | | 0.87 | 0.02 (0.03) | | 1.02 |
| Afghanistan | -0.96 (0.10) | | 0.38 | -0.07 (0.04) | | 0.93 |
| Poland | 0.24 (0.07) | | 1.27 | -0.46 (0.11) | | 0.63 |
| Bosnia and Herzegovina | 0.30 (0.06) | | 1.35 | 0.16 (0.07) | | 1.17 |
| Romania | 0.14 (0.08) | | 1.15 | 0.26 (0.06) | | 1.30 |
| Croatia | 0.23 (0.08) | | 1.26 | 0.18 (0.08) | | 1.19 |
| North Macedonia | 0.20 (0.10) | | 1.22 | 0.16 (0.08) | | 1.18 |
| Hungary | 0.31 (0.11) | | 1.36 | 0.30 (0.10) | | 1.34 |
| Slovakia | 0.19 (0.13) | | 1.21 | -0.06 (0.11) | | 0.95 |
| Egypt | -0.12 (0.12) | | 0.89 | -0.03 (0.13) | | 0.97 |
| Bulgaria | -0.01 (0.14) | | 0.99 | 0.10 (0.13) | | 1.11 |
| Iran | -0.28 (0.13) | | 0.76 | -0.14 (0.14) | | 0.87 |
| Iraq | -0.20 (0.15) | | 0.82 | 0.00 (0.13) | | 1.00 |
| Tunisia | -0.43 (0.22) | | 0.65 | 0.01 (0.15) | | 1.01 |
| Czech Republic | -0.09 (0.21) | | 0.91 | -0.36 (0.23) | | 0.70 |
| Syria | -0.16 (0.19) | | 0.85 | -0.20 (0.21) | | 0.82 |
| *Interaction: female x countries* |  | |  |  | |  |
| Serbia | -0.19 (0.05) | | 0.83 | -0.04 (0.05) | | 0.96 |
| Turkey | -0.50 (0.05) | | 0.61 | -0.34 (0.05) | | 0.71 |
| Afghanistan | -0.59 (0.16) | | 0.55 | -0.48 (0.16) | | 0.62 |
| Poland | -0.27 (0.10) | | 0.77 | -0.19 (0.10) | | 0.83 |
| Bosnia and Herzegovina | -0.36 (0.10) | | 0.70 | -0.29 (0.10) | | 0.75 |
| Romania | -0.23 (0.11) | | 0.80 | -0.05 (0.11) | | 0.95 |
| Croatia | 0.05 (0.12) | | 1.05 | 0.06 (0.12) | | 1.06 |
| North Macedonia | -0.57 (0.15) | | 0.57 | -0.36 (0.15) | | 0.70 |
| Hungary | 0.07 (0.14) | | 1.08 | 0.29 (0.14) | | 1.34 |
| Slovakia | -0.32 (0.16) | | 0.73 | -0.09 (0.16) | | 0.91 |
| Egypt | -0.39 (0.22) | | 0.68 | -0.26 (0.22) | | 0.78 |
| Bulgaria | 0.02 (0.20) | | 1.02 | 0.21 (0.20) | | 1.23 |
| Iran | -0.14 (0.27) | | 0.87 | -0.34 (0.27) | | 0.71 |
| Iraq | -0.61 (0.30) | | 0.55 | -0.56 (0.30) | | 0.57 |
| Tunisia | -0.22 (0.32) | | 0.81 | 0.01 (0.32) | | 1.01 |
| Czech Republic | 0.00 (0.28) | | 1.00 | 0.10 (0.28) | | 1.11 |
| Syria | -1.92 (0.61) | | 0.15 | -1.60 (0.61) | | 0.20 |
| Likelihood ratio test | 700 (38df) | | | 3,577 (70df) | | |

Source: LMDB. *Int*: Interaction. Model M Nationality net includes individual and occupational predictors and controls (see Table 1).
