# Supplementary material for: The impact of occupational structures on ethnic and gendered employment gaps: An event history analysis using social security register data
Source: PLoS One. 2021 Apr 15;16(4):e0250398. doi: 10.1371/journal.pone.0250398 (PMC8049483; doi:10.1371/journal.pone.0250398)
Supplement: S4 Table — (DOCX) [file pone.0250398.s005.docx]

S4 Table: Youth unemployment rates in Vienna (18-28-year olds)

| Year | Natives | Immigrants | All |
| --- | --- | --- | --- |
| 2011 | 11.2 | 14.2 | 12.1 |
| 2012 | 11.8 | 15.4 | 12.9 |
| 2013 | 12.9 | 16.1 | 13.9 |
| 2014 | 13.6 | 17.3 | 14.8 |
| 2015 | 13.8 | 19.7 | 15.8 |
| 2016 | 13.1 | 20.6 | 15.7 |
| 2017 | 12.3 | 19.8 | 14.9 |
| 2018 | 10.9 | 17.9 | 13.3 |

Source: Statistics Austria, Register-based Labour Market Statistics, retrieved from STATcube - Statistical Database of Statistics Austria (http://www.statistik.at/web_en/publications_services/statcube/index.html).
