# Supplementary material for: The impact of occupational structures on ethnic and gendered employment gaps: An event history analysis using social security register data
Source: PLoS One. 2021 Apr 15;16(4):e0250398. doi: 10.1371/journal.pone.0250398 (PMC8049483; doi:10.1371/journal.pone.0250398)
Supplement: S5 Table — (DOCX) [file pone.0250398.s006.docx]

S5 Table: Sensitivity analysis: stratified cox proportional hazards, time to recurrent employment events

|  | R1: top-quintile immigrant jobs excluded | | | R2: females only | | | R3: males only | | |
| --- | --- | --- | --- | --- | --- | --- | --- | --- | --- |
|  | b (SE) | hazard | | b (SE) | hazard | | b (SE) | hazard | |
| *Gender (ref: male)* |  |  | |  |  | |  |  | |
| Female | -0.06 (0.05) | | 0.94 |  | |  |  | |  |
| *Int*: female x time (/100 days) | -0.03 (0.01) | | 0.97 |  | |  |  | |  |
| *Education (ref: lower secondary)* |  | |  |  |  | |  | |  |
| No lower secondary certificate | -0.54 (0.12) | | 0.58 | -0.42 (0.11) | | 0.66 | -0.36 (0.07) | | 0.69 |
| Apprenticeship | 0.33 (0.05) | | 1.39 | 0.41 (0.06) | | 1.50 | 0.30 (0.05) | | 1.35 |
| VET school | 0.58 (0.06) | | 1.79 | 0.65 (0.07) | | 1.91 | 0.55 (0.07) | | 1.74 |
| Academic School (Gymnasium) | 0.42 (0.06) | | 1.52 | 0.53 (0.07) | | 1.71 | 0.35 (0.07) | | 1.42 |
| VET college | 0.69 (0.05) | | 1.99 | 0.83 (0.06) | | 2.28 | 0.58 (0.06) | | 1.80 |
| University | 0.84 (0.04) | | 2.32 | 0.88 (0.05) | | 2.41 | 0.80 (0.06) | | 2.23 |
| *Work experience in 2013 (ref: none)* |  | |  |  | |  |  | |  |
| Up to 90 days | 0.29 (0.04) | | 1.34 | 0.32 (0.04) | | 1.38 | 0.38 (0.04) | | 1.46 |
| 91-180 days | 0.43 (0.04) | | 1.54 | 0.45 (0.05) | | 1.57 | 0.57 (0.04) | | 1.77 |
| More than 180 days | 0.62 (0.03) | | 1.85 | 0.63 (0.03) | | 1.88 | 0.75 (0.03) | | 2.12 |
| *Structural variables* |  | |  |  |  | |  | |  |
| Occupational closure M (z) | 0.07 (0.03) | | 1.08 | 0.16 (0.03) | | 1.17 | 0.10 (0.02) | | 1.11 |
| *Int*: M x female | 0.09 (0.04) | | 1.10 |  | |  |  | |  |
| *Int*: M x female x time (/100 days) | -0.03 (0.01) | | 0.97 | -0.03 (0.01) | | 0.97 |  | |  |
| Ethnic segregation (% foreign empl.) | -0.15 (0.07) | | 0.86 | -0.17 (0.04) | | 0.84 | -0.02 (0.03) | | 0.98 |
| *Int*: Ethnic segregation x female | -0.19 (0.09) | | 0.83 |  | |  |  | |  |
| Gender segregation (% female empl.) | 0.02 (0.03) | | 1.02 | -0.10 (0.04) | | 0.90 | 0.01 (0.02) | | 1.01 |
| *Int*: Gender segregation x female | -0.14 (0.06) | | 0.87 |  | |  |  | |  |
|  |  | |  |  | |  |  | |  |
| *Individual controls* | yes | |  | yes | |  | yes | |  |
| *Origin countries* | yes | |  | yes | |  | yes | |  |
| Likelihood ratio test | 2,011 (70df) | | | 1,870 (46df) | | | 1,611 (45df) | | |

Source: LMDB. *Int*: Interaction. Individual controls: age group, years in country, marital status. Country controls are country dummies including gender interactions (R1). Unemployment-vacancy ratio is included as structural level control. R1: n = 69,617, number of events = 9,865; R2: n = 52,425, number of events = 6,321; R3: n = 60,276, number of events = 8,539.
