# Supplementary material for: The impact of occupational structures on ethnic and gendered employment gaps: An event history analysis using social security register data
Source: PLoS One. 2021 Apr 15;16(4):e0250398. doi: 10.1371/journal.pone.0250398 (PMC8049483; doi:10.1371/journal.pone.0250398)
Supplement: Attachment — Submitted filename: Response-to-Reviewers.docx [file pone.0250398.s007.docx]

Dear Prof Rosenbloom!

Please find below our response to the very valuable and helpful comments of you and the 2 reviewers.

Following your suggestion, we have tried to make it clear very early in the introduction (lines 43-53) what the article is going to do empirically. This was missing of course, we hope this facilitates reading the article. We have also tried to make the theoretical framework more specific by referring to our empirical problem. Please read more on that below in our response to the reviewers, especially to reviewer 1.

Response to Reviewer 1

Your point about the significance of the occupational characteristics of the “desired job” is a very good one. We address this at the beginning of the data section “Structural occupational variables” (p. 13 lines 285 - 296) where we highlight its importance for the job search process. We have changed the label to “aspired job” throughout the paper, because we believe it better reflects its purpose in the job search, although “desired job” would be the correct translation of “Berufswunsch”, which is the German term used by the PES. The PES focusses most if not all of its job search assistance on this job, which is not a mere “desire” of the individual job searcher but is closely related to the education and the prior experience as cleared with the PES case worker (it is thus also a “realistic” job to search for where the prospects are best given individual characteristics and of course also demand).

We considerably revised the theoretical section on the mechanisms underlying the structural effects in order to remedy the concerns raised. Our central argument about occupational specificity is the following: like job searchers’ aspired job belongs to a particular occupational group, these occupational groups demand more or less specific credentials. Occupational specificity thus limits competition for these jobs in the sense that it at least to some extent shelters those applicants who possess such credentials from competition from other applicants who do not have a preferred credential or a less preferred one. Except for the regulated professions however, very different credentials can apply for most jobs and we see empirically that this is the case. Now if the occupational specificity is low, competition is high as there seems to be no preferred credential but applicants from diverse educational backgrounds have good chances and actually do get such jobs. These tend to be the low-paying and low-prestige jobs (except of course managerial jobs who also have diverse educational backgrounds) and if so, they tend to be populated disproportionally by immigrants and/or females. Because the %foreign and %female structural effect mainly or exclusively applies to females (males tend to benefit from ethnic segregation and there is no effect for males regarding %females) the question about the mechanism then is why immigrant females do benefit from looking for a job in occupations with less immigrants and to some extent also in occupations with less females. We agree that this contradicts the assumption that employers deem them to be unsuitable for the job. However, we now argue more clearly that this may be outweighed by the fact that immigrants tend to apply for immigrant jobs which are located at the bottom of the labour market and where they are subject to fierce competition. In line with the queuing argument, employer may privilege native workers in order to uphold a minimum of diversity. At the top of the occupational ladder this may be the other way around: on top of less competition, skilled minorities may enjoy opportunities in order to increase diversity (p. 8-9, lines 168-190).

Likewise, we have expanded and refined our argument regarding the gendered structure thanks to your suggestions (p. 9-10, lines 205-216).

We continue to use the term “employment gap” throughout the paper but as we agree that the time-to-employment dimension is really useful, we emphasized this dimension in several parts throughout the article.

You are correct regarding Figure 2, the baseline represents the average person in the pooled data. We decided to stick with this and mentioned on p.21 lines 453-456 that “The gender differences in the incidence rates reflect the fact that the average man finds a new job faster than the average woman.”

As detailed on p. line occupational labour markets are an institutional feature of some countries, especially in continental European countries, while industrial relations in others countries, e.g. in more liberal employment regimes credentials attained in secondary schooling are of less relevance with a relatively loose linkage between schooling and work.

Thank you for noticing the error in phrasing H2, which we corrected.

Response to Reviewer 2

We provided additional context information to aid interpretation. In the supplement (S4 table) we present recent trends in youth unemployment among our age-group (18-28 year olds) and for natives and immigrants as well. We included a text that refers to this information on p.10-11 lines 229-234. We also mention how gender and ethnically segregated the labour market actually is by referring to S2 Table (p. 14, lines 314-316).

We have also included another supplemental table (S5 table) that displays the results of a sensitivity analysis following your suggestions. We ran one model that excludes occupations with very high shares of immigrant workers (more specifically we excluded those 9 occupational groups who are in the top quintile in the immigrant share, i.e. > 48%). Two additional models were run using the data of women and men, respectively, only. We discuss these results on p.23 lines 509-515. As the results of these models are very much in line with the main findings we base our article on, we are confident that they are quite robust.

The remark about how gender norms operate is similar to the issue raised by R1. In response, we have considerably refined and extended our argument following much of your very valuable suggestions p. 9-10, lines 205-216 and in the results section on p. 17, lines 394-398 and in the discussion section on p.23, lines 522-527. Thank you for suggesting the blue-collar and white-collar distinction which we also took up not by referring to Charles and Grusky (this very interesting book seems to be unavailable in AT, even Vienna University does not hold a copy, but it is on its way from Amazon and I am looking forward to reading it) but by referring to other articles that make a similar case by following Charles/Grusky themselves.

We have also included a remark about the transferability of the credentials on p.12 lines 270-271. Our data provides information on the educational credential that is deemed to be marketable by the PES case worker.

Your valuable comments and suggestions have inspired us to be more specific in our theoretical argument as well as the discussion of our results which complements our discussion about the functioning of the Viennese labour market in p.24-25 lines 533-566.
