# Supplementary material for: The impact of occupational structures on ethnic and gendered employment gaps: An event history analysis using social security register data
Source: PLoS One. 2021 Apr 15;16(4):e0250398. doi: 10.1371/journal.pone.0250398 (PMC8049483; doi:10.1371/journal.pone.0250398)
Supplement: Attachment — Submitted filename: Response-to-Reviewers-R2.docx [file pone.0250398.s008.docx]

Response to Reviewer 1

We acknowledge that there may be an apparent inconstancy in the development of hypothesis 2. It is true that the two sentences lead to the expectation that native and foreign workers are affected differently by ethnic occupational segregation:

“Indeed, in occupations with a large share of immigrant workers employers may privilege the occasional native applicant when there is a premium on a minimum of diversity. The same may apply to jobs at the top where most of the workers are natives and minority workers may be welcome in order to increase diversity.”

However, while the former mechanism may occur occasionally, the latter should outweigh it by far. To clarify this we add the sentence:

“Still, as native workers have largely abandoned the jobs increasingly held by immigrants, the first diversity argument is likely insignificant in explaining aggregate labour market outcomes, while the second one has much more relevance because occupations predominantly held by natives do offer better employment prospects.” (l184-187).

So, in fact we expect the effect as stated in Hypothesis 2, which, as we see only holds true for women. We follow your suggestion of including an interaction term of “foreigner” and “% foreign employees” as an additional robustness test. It yields a positive coefficient which is statistically insignificant (so we see no need to include it in the main paper). However, we prepared a supplementary figure based on this model (S6 figure), which suggests that the observation that male workers benefit form ethnic segregation (contrary to H2) seems to be driven mostly by foreign workers who tend to benefit more than native workers. This is discussed and described in lines 483-494.

Response to Reviewer 2

Your suggestion to include a final figure could in principle aid comprehension of the relative importance of the different factors. You are right that the two human capital variables (education and work experience) are the main drivers of reemployment probabilities. However, we would not want to include such an additional figure because our main concern are the structural effects over and above the individual human capital characteristics and thus we want to limit graphical depictions on differences in the incidence ratios related to those variables. These differences, although certainly not as large as between the highest and the lowest education level, remain the same among persons with the same education or work experience. The fact that education and work-experience have larger effects is nicely illustrated by the hazard ratios in table 1 as well as in the text. The same applies to their relative contribution in terms of model fit, as can be seen in the Likelihood Ratio tests in Table 1
